# Mechanistic insight into the anti-inflammatory and lung-protective effects of *Agrimonia pilosa* extract via NF-κB/MAPK inhibition in ovalbumin- and lipopolysaccharide-induced respiratory inflammation models

**DOI:** 10.1080/13880209.2026.2627661

**Published:** 2026-03-01

**Authors:** Yeong-Geun Lee, Jeong Eun Kwon, Dae Won Park, Hae Rim Lee, Jinhyuk Lee, Yong-Min Choi, Eun-Ji Cho, Se Chan Kang

**Affiliations:** ^a^ Department of Oriental Medicine Biotechnology and Graduate School of Biotechnology, Kyung Hee University, Yongin, Gyeonggi, Republic of Korea; ^b^ BioMedical Research Institute, Kyung Hee University, Yongin, Republic of Korea; ^c^ Daesang Wellife, Seoul, Republic of Korea; ^d^ Research Institute, Mbiometherapeutics Co., Ltd, Yongin, Gyeonggi, Republic of Korea

**Keywords:** Respiratory system, anti-inflammation, NF-κB, *Agrimonia pilosa*

## Abstract

**Context:**

Asthma and COPD involve airway inflammation, oxidative stress, and epithelial cell apoptosis. While corticosteroids are common, long-term use can cause adverse effects, prompting interest in plant-based anti-inflammatory alternatives. *Agrimonia pilosa* (AP) shows promise, but its effectiveness in airway inflammation requires further study.

**Objective:**

To assess anti-inflammatory and lung-protective effects in LPS-stimulated A549 cells and an OVA+LPS mouse model, focusing on NF-κB/MAPK pathways and apoptosis-related markers.

**Materials and methods:**

A549 cells were pretreated with AP and stimulated with LPS. Pro-inflammatory cytokine mRNA levels and phosphorylation of NF-κB p65, p38, ERK, JNK were measured. *In vivo*, AP was given orally during OVA+LPS challenges. Cytokines and chemokines in bronchoalveolar lavage fluid (BALF), lung matrix metalloproteinases (MMP-1/9/12), Bax/Bcl-2 ratio, histopathological analysis, and systemic toxicity markers (AST, ALT, ALP, and BUN), body and organ weights, and gross examination were evaluated.

**Results:**

In A549 cells, AP reduced LPS-induced pro-inflammatory mRNA and inhibited NF-κB p65 and MAPK phosphorylation. In the OVA+LPS model, oral AP lowered BALF levels of CXCL-1, CXCL-2, IL-1β, IL-6, and TNF-α, and downregulated lung MMP-1/9/12, reducing Bax/Bcl-2 ratio with histological improvements. At 100 mg/kg, CXCL-1 and CXCL-2 decreased to about 73.2% and 89.2%, respectively. IL-1β and IL-6 levels decreased by 72.5% and 37.4%, respectively, with IL-6 significant only at the high dose. No systemic toxicity was observed, with stable serum toxicity markers and no abnormal findings.

**Discussion and conclusions:**

AP exhibits anti-inflammatory and lung-protective effects by inhibiting NF-κB/MAPK signaling and regulating proteolysis and apoptosis, indicating it as a safe, effective treatment for airway inflammation.

## Introduction

The respiratory system plays a crucial role in maintaining homeostasis by facilitating the uptake of oxygen and the removal of carbon dioxide. Gas exchange occurs in the alveoli, where oxygen is transported to tissues for ATP production, while carbon dioxide, a metabolic byproduct, is expelled to regulate body pH (Kaminsky et al. 2023). However, due to a lack of protective barriers such as mucus and cilia, alveoli are vulnerable to environmental insults, and alveolar macrophages play a key role in immune defense.

Chronic respiratory diseases such as asthma and chronic obstructive pulmonary disease (COPD) are characterized by airway inflammation, mucus hypersecretion, and airway remodeling. These diseases can be exacerbated by environmental factors such as pollutants and allergens, and are increasing in prevalence, particularly among the elderly (Ko et al. 2020; Luo et al. 2020; Zhang et al. 2020). Asthma is driven by allergic inflammation, while COPD is closely associated with smoking and aging (An et al. 2012; Wang et al. 2020).

Both asthma and COPD are inflammatory lung diseases, and the nuclear factor kappa B (NF-κB) and mitogen-activated protein kinase (MAPK) signaling pathways are known to play key roles in their pathogenesis (Gu et al. 2017). NF-κB is a transcription factor that regulates the expression of pro-inflammatory cytokines and is activated by stimuli such as tumor necrosis factor alpha (TNF-α) and interleukin-1 beta (IL-1β). In airway epithelial cells of patients with chronic or allergic inflammation, activation of NF-κB leads to secretion of inflammatory mediators, recruitment of alveolar macrophages and neutrophils, and release of proteases and reactive oxygen species, all of which can damage lung tissue (Su et al. 2011).

The MAPK pathway transduces extracellular inflammatory signals into intracellular responses *via* a kinase cascade (Wang et al. 2018). Its major components (p38, ERK, and JNK) are phosphorylated during inflammation, leading to expression of pro-inflammatory cytokines (Santana et al. 2020).

Oxidative stress further exacerbates inflammation by stimulating alveolar macrophages to secrete mediators such as TNF-α, interleukins (ILs), and chemokines such as the C-X-C motif ligand (CXCL)-1 and CXCL-2. These chemokines promote recruitment of neutrophils and monocytes, perpetuating inflammatory responses (Yang et al. 2012; Lee and Yang 2013; Barnes 2018). IL-1β is associated with severe inflammation, IL-6 supports eosinophil migration and survival, and TNF-α contributes to chronic neutrophilic inflammation (Yamaoka et al. 1998; Siomek 2012). Mucin 5ac (MUC5AC) is a prominent glycoprotein found in human airway mucus. Numerous studies have indicated that its aberrant expression plays a critical role in the development of respiratory diseases (Chen et al. 2025).

Activated immune cells also secrete matrix metalloproteinases (MMPs), such as MMP-1, MMP-9, and MMP-12, which degrade components of the extracellular matrix and disrupt lung tissue structure (Nakagome and Nagata 2011). Neutrophil-mediated overexpression of MMP-9 further increases protease activity and reduces antiprotease defenses, contributing to lung destruction and fibrosis (Abboud and Vimalanathan 2008; Barnes 2010).

Chronic inflammation can lead to apoptosis, or programmed cell death, of lung epithelial cells. This process is regulated by caspases and mitochondrial pathways involving proteins of the Bcl-2 family. An increased Bax/Bcl-2 ratio promotes the release of cytochrome C from mitochondria, activating the caspase cascade and leading to apoptosis (Porębska et al. 2006). Considering that oxidative stress and inflammation are mechanistically interconnected and predominantly regulated *via* NF-κB/MAPK signaling in chronic airway diseases (Rahman and Adcock 2006; Schuliga 2015), therapeutic candidates that can simultaneously reduce inflammatory mediator production and restore redox balance are particularly noteworthy. Consequently, plant-derived agents demonstrating integrated anti-inflammatory and antioxidant properties are under active investigation as safer therapeutic alternatives or adjunct treatments to current therapies.

*Agrimonia pilosa* Ledeb. (AP) is a perennial herbaceous plant widely distributed across Korea, China, Japan, and India. Traditionally used in East Asian medicine for its anti-inflammatory properties, it has recently attracted attention as a source of bioactive compounds (Hsu et al. 1987; Bae et al. 2010). Studies have reported various pharmacological activities, including antioxidant (Zhu et al. 2009; Kato et al. 2010), antitumor (Koshiura et al. 1985), antiviral (Shin et al. 2010), antibacterial (Yamaki et al. 1989), hypoglycemic (Jung et al. 2006), and anti-inflammatory effects (Jung et al. 2010). However, its effects on airway inflammation remain unclear. Based on our previous studies, a 50% ethanolic extract of *A. pilosa* exhibited potent inhibitory activity against influenza A and SARS-CoV-2 viruses (Lee et al. 2021; Joo et al. 2022). Additionally, apigenin-7-glucuronide was identified as the major component based on qualitative (LC/MS) and quantitative (HPLC) analyses (Kwon et al. 2019; Park et al. 2020). In this investigation, an aqueous extract was prepared to enhance its suitability for repeated oral administration and to support traditional applications. Additionally, apigenin-7-glucuronide was quantified as a marker compound to standardize the extract.

In this study, we evaluated the anti-inflammatory and lung-protective effects of *A. pilosa* extract using a murine model induced by ovalbumin (OVA) and lipopolysaccharides (LPS) to simulate key features of asthma and COPD. We aimed to determine its regulatory effects on inflammatory mediators, MMPs, and apoptosis-associated pathways, thereby investigating its potential as a plant-derived candidate for the treatment of airway inflammatory diseases. It is hypothesized that AP mitigates airway inflammation and lung injury through the suppression of NF-κB/MAPK activation, leading to a decrease in inflammatory mediators, MMP expression, and markers related to apoptosis.

## Materials and methods

### Chemicals

OVA (Cat No. A5503) and LPS (Cat No. L2880) were obtained from Sigma-Aldrich (St. Louis, MO, USA). TRIzol was purchased from Invitrogen (10296010, Carlsbad, CA, USA). Dexamethasone (DEX) was obtained from TCI (D1961, Tokyo, Japan), while Al(OH_3_) was purchased from InvivoGen (vac-alu-50, San Diego, CA, USA). RNAiso Plus was sourced from TaKaRa Bio (9109, Otsu, Japan).

### Preparation of Agrimonia pilosa

The dried aerial parts of AP were purchased from BioKorea (Seoul, Korea). A voucher specimen (BMRI-AP-1601) was deposited at the Bio-Medical Research Institute of Kyung Hee University, Yongin, Korea. Hot-water extraction was selected to reflect traditional oral usage and to enhance translational feasibility for repeated oral administration by eliminating residual organic solvents, while also capturing a broad spectrum of polar constituents, including flavonoid glucuronides such as apigenin-7-glucuronide. AP was extracted with purified water at 90 ± 5 °C for 6 h and filtered. The extraction yield was 14.6% (w/w, dried extract weight per dried *A. pilosa*). After concentrating the extract under reduced pressure, the AP extract was freeze-dried and stored at 4 °C until use.

### HPLC quantitative analysis

A calibration curve for apigenin 7-glucuronide was constructed using five concentrations (10–160 μg/mL) prepared in 50% methanol. For each injection, a 5 μL aliquot of the extract solution was analyzed using an Agilent 1260 Infinity series HPLC system (Agilent Technologies Inc., Santa Clara, CA, USA) equipped with a G7115A 1260 DAD WR detector set at 320 nm. Chromatographic separation was achieved using a Capcell PAK C18 column (5 μm, 250 × 4.6 mm; Osaka Soda Co., Ltd., Tokyo, Japan). The mobile phase consisted of 0.1% phosphoric acid in water (solvent A) and acetonitrile (solvent B). The gradient elution for solvent B was programmed as follows: 20% at 0.01 min, increased to 60% at 10 min, then to 100% at 11 min, maintained until 17 min, returned to 20% at 18 min, and held until 25 min. The flow rate was maintained at 0.8 mL/min. Each analytical run was performed in triplicate to ensure precision.

### Cell culture and cytotoxicity assay

Cells from the human non-small cell lung cancer (NSCLC) cell line A549 were purchased from Korean Cell Line Bank (KCLB No. 10185, Seoul, Korea) and cultured in RPMI-1640 medium (10–040, Corning Inc., New York, NY, USA) supplemented with 10% fetal bovine serum (Corning Inc.) and 1% penicillin-streptomycin (Cat. No.50-195-9713, GenDEPOT Inc., Baker, TX, USA). Cells were maintained in a humidified incubator at 37 °C with 5% CO_2_.

To evaluate the cytotoxicity of AP, A549 cells (3 × 10^4^ cells/well) were seeded in 96-well plates and incubated overnight. Cells were then treated with AP at concentrations of 12.5, 25, 50, and 100 μg/mL. After 24 h, an MTT solution was added to each well and incubated for 2 h. The medium was removed, and dimethyl sulfoxide was added to dissolve the formazan crystals. Absorbance was measured at 595 nm using an Infinite 200 microplate reader (TECAN, Zurich, Switzerland). To assess cytotoxicity under inflammatory conditions, cells were pretreated with various concentrations of AP for 1 h, followed by LPS stimulation (10 μg/mL) for 24 h. The subsequent steps, including MTT staining and absorbance measurements, were performed as described above.

### Quantitative real-time PCR analysis

Total RNA was isolated using TRIzol reagent (Invitrogen), following the manufacturer’s instructions. Reverse transcription of 2 μg of isolated RNA was conducted using the PrimeScript ІІ 1st Strand cDNA Synthesis Kit (Takara, Japan). The mRNA levels of the target genes were assessed using SYBR Premix Ex Taq (Takara, Japan). The quantitative real-rime PCR (qRT-PCR) was performed using a QuantStudio3 Real-Time PCR system (Thermo Fisher Scientific., USA). The primer sequences used in the experiments are listed in Table 1. The thermal cycling conditions consisted of an initial denaturation step at 95 °C for 10 min, followed by 40 cycles of denaturation at 95 °C for 15 s and 60 °C for 60 s. Relative mRNA levels were determined using the comparative cycle threshold method. Data resulting from qRT-PCR were normalized to the *β-actin* housekeeping gene, and expressed as a ratio relative to the value of the untreated group.

**Table 1.** Primer sequences used in qRT-PCR.

Gene name	Prime sequence
Human	
IL-1β	5′-ACCTGTGTCTTTCCCGTGG-3′ (sense) 5′-TCATCTCGGAGCCTGTAGTG-3′ (antisense)
IL-6	5′-GGAGAGGAGACTTCACAGAGGA-3′ (sense) 5′-ATTTCCACGATTTCCCAGAGA-3′ (antisense)
TNF-α	5′-CCTATGTCTCAGCCTCTTCTCAT-3′ (sense) 5′-CACTTGGTGGTTTGCTACGA-3′ (antisense)
iNOS	5′-CACAGAACTGAGGGTACA-3′ (sense) 5′-AGAGAGATCGGGTTCACA-3′ (antisense)
COX-2	5′-AGATCACCTCTGCCTGAGTA-3′ (sense) 5′-TTA AAATGAGATTGTCCGAA-3′ (antisense)
β-actin	5′-TAAAACGCAGCTCAGTAACAGTCGG-3′ (sense) 5′-TGCAATCCTGTGGCATCCATGAAAC-3′ (antisense)
Mouse	
MMP-1	5′-TTGCCCAGAGAAAAGCTTCA-3′ (sense) 5′-TAGCAGCCCAGAGAAGCAAC-3′ (antisense)
MMP-2	5′-GATAACCTGGATGCCGTCGTG-3′ (sense) 5′-CTTCACGCTCTTGAGACTTTG-3′ (antisense)
MMP-9	5′-GCCCTGGAACTCACACGACA-3′ (sense) 5′-TTGGAAACTCACACGCCAGAAG-3′ (antisense)
MMP-12	5′-GCTAGAAGCAACTGGGCAAC-3′ (sense) 5′-ACCGCTTCATCCATCTTGAC-3′ (antisense)
Bax	5′-CTACAGGGTTTCATCCAG-3′ (sense) 5′-CCAGTTCATCTCCAATTCG-3′ (antisense)
Bcl-2	5′-GTGGATGACTGAGTACCT-3′ (sense) 5′-CCAGGAGAAATCAAACAGAG-3′ (antisense)
β-actin	5′-CGTGAAAAGATGACCCAGAT-3′ (sense) 5′-ACCCTCATAGATGGGCACA-3′ (antisense)
β-actin	5′-CGTGAAAAGATGACCCAGAT-3′ (sense) 5′-ACCCTCATAGATGGGCACA-3′ (antisense)

### Western blot analysis

A549 cells (5 × 10^5^ cells/well) were seeded into 60 mm culture dishes and incubated overnight. Cells were then treated with AP (25, 50, and 100 μg/mL) for 1 h, followed by LPS (10 μg/mL) stimulation for 6 h. After incubation, cells were lysed in a RIPA buffer (Sigma-Aldrich) containing a protease inhibitor cocktail, and the lysates were incubated on ice for 30 min with intermittent vortexing. The lysates were then centrifuged at 14,000 rpm for 20 min at 4 °C to collect the supernatant. Total protein (50 μg) was mixed with a 2× Laemmil sample buffer (Sigma-Aldrich) and denatured at 95 °C for 10 min. Proteins were separated by sodium dodecyl sulfate-polyacrylamide gel electrophoresis and transferred onto a polyvinylidene difluoride membrane. After blocking with 5% skim milk in tris-buffered saline and Tween 20, membranes were incubated with primary antibodies, followed by secondary antibodies. Protein expression was visualized to evaluate target protein levels.

### Experimental animals sample size

Six-week-old male Balb/c mice (20–22 g) were obtained from Orient Bio Inc. (Korea). All animals were randomly divided into 6 groups (5 in each group). The sample size was determined based on previous studies conducted under similar experimental conditions, in which 5 mice per group were sufficient to detect significant differences among treatment. Animals of the same age and sex were used to minimize biological variation, and their number was restricted to the minimum required by ethical. The mice were acclimated for 1 week prior to the experiment in a controlled environment (room temperature, 25 ± 1 °C; relative humidity, 50 ± 10%; 12 h light/dark cycle). All experimental procedures were approved by the Institutional Animal Care and Use Committee of Kyung Hee University (KHUASP-23-252) and were conducted from June 29^th^, 2023, to June 28^th^, 2024.

### OVA+LPS-induced airway inflammation mouse model

**Figure 1.**
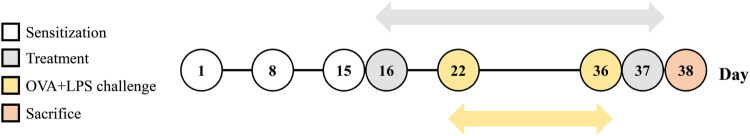
Experimental protocol for the development of respiratory disease models. Schematic diagrams for the experi­ments of OVA+LPS-induced mouse model.

All animals were randomly assigned to six groups (*n* = 5 per group), and the experimental timeline is shown in Figure 1. Briefly, mice were sensitized *via* intraperitoneal injection on days 1, 8, and 15 (at the same time of day) with 50 μL of a sensitizing mixture prepared by combining OVA (1 mg/mL) and aluminum hydroxide [Al(OH)_3_, 10 mg/mL] at a 1:1 (*v/v*) ratio (final concentrations: OVA 0.5 mg/mL and Al(OH)_3_ 5 mg/mL; dose per mouse: OVA 25 μg and Al(OH)_3_ 250 μg), followed by intraperitoneal injection of 50 μL of LPS (50 μg/mL; dose per mouse: 2.5 μg) with a 10 mins interval between injections. From day 16 to day 37, mice received AP (25, 50, or 100 mg/kg) or dexamethasone (DEX, 2 mg/kg) dissolved in PBS by oral gavage once daily at a consistent time each day. The DEX dose (2 mg/kg) was utilized as a positive control, in accordance with previously published murine airway inflammation and asthma studies that documented significant anti-inflammatory effects at the same dosage (Guihua et al. 2016; Xu et al. 2017). During the challenge period, AP and DEX were administered 1 h prior to each OVA+LPS challenge. Airway inflammation was induced by intranasal instillation every other day from day 22 to day 36 (at the same time of day) using 30 μL of a freshly prepared mixture of OVA (1 mg/mL) and LPS (50 μg/mL) at a 1:1 (*v/v*) ratio (final concentrations: OVA 0.5 mg/mL and LPS 25 μg/mL; dose per challenge: OVA 15 μg and LPS 0.75 μg). All mice were euthanized on day 38 by inhalation of 0.5 ∼ 1.0% isoflurane in accordance with the approved animal protocol, and samples were collected for subsequent analyses.

### Serum analysis

Blood samples were centrifuged at 3,000 *g* for 10 min at 4 °C, and the supernatant was transferred to a 1.5-mL microtube for serum collection. Serum analysis was performed using commercial kits according to the manufacturer’s instructions (MyBioSource, Sandiego, CA, USA). Serum triglyceride (TG: MBS9716974), total cholesterol (Total C: MBS269999), high-density lipoprotein cholesterol (HDL-C: MBS268119), and low-density lipoprotein cholesterol (LDL-C: MBS706909) levels were measured. Liver function markers, including asparatate aminotransferase (AST: MBS265501), alanine aminotransferase (ALT: MBS264717), and alkaline phosphatase (ALP: MBS264223), as well as blood urea nitrogen (BUN: MBS2611085), were also determined. All absorbance measurements were performed using a microplate reader (Spark 10 M, Tecan, Switzerland).

### Bronchoalveolar lavage fluid

After blood collection, bronchoalveolar lavage fluid (BALF) was collected by instilling and retrieving 1 mL of cold sterile saline into the lung tissues. This process was performed in triplicate (Abdelaziz et al. 2018). The collected BALF was centrifuged at 1,500 *g* for 10 min at 4 °C, and the supernatants were stored at −80 °C until further use.

### Enzyme-linked immunosorbent assay

The levels of CXCL-1, CXCL2, IL-1β, IL-6, and TNF-α production in BALF were measured using an enzyme-linked immunosorbent assay (ELISA) according to a protocol provided by Boster Bio (Pleasanton, CA, USA).

### Lung histopathology

After the collection of BALF, lung tissues were excised, fixed with 4% paraformaldehyde for 24 h, dehydrated, embedded in paraffin, and cut into 5-μm sections. The sections were then stained with hematoxylin and eosin (H&E) to evaluate inflammation.

### Statistical analysis

All results are presented as mean ± standard error of the mean (SEM). For *in vitro* experiments, data were derived from three independent assays. For *in vivo* experiments, each group was composed of five mice (*n* = 5), with each mouse serving as an independent biological replicate. When assays necessitated replicate measurements, samples from each mouse were measured in technical triplicates and averaged to produce a single value per mouse for statistical evaluation. Statistical significance was assessed using a one-way analysis of variance followed by Tukey’s post hoc test. A P-value of less than 0.05 was regarded as statistically significant. Data analysis was conducted utilizing GraphPad Prism 5 (GraphPad Software Inc.).

## Results

### Quantitative analysis of apigenin-7-glucuronide in AP

**Figure 2.**
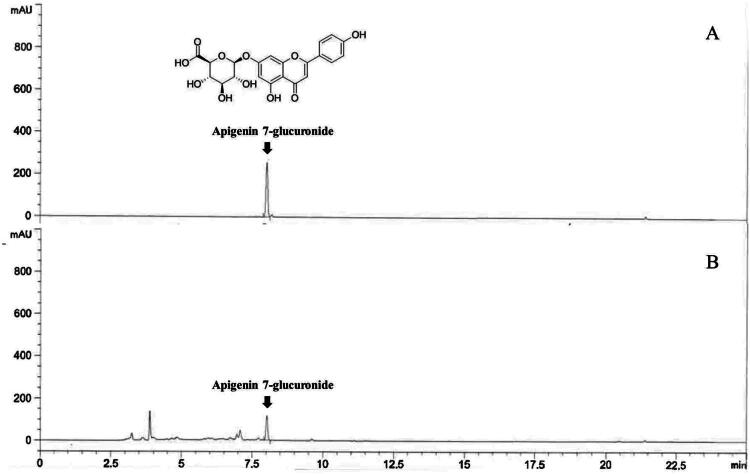
HPLC chromatogram of *Agrimonia pilosa* water extract (AP). (A) standard chromatogram of apigenin 7-glucuronide (80 μg/mL). (B) Representative chromatogram of AP (10 mg/mL).

The water extract of AP was standardized, with apigenin-7-glucuronide revealed as a primary constituent in our prior research, based on HPLC quantitative analysis (Kwon et al. 2019; Park et al. 2020). The quantification of apigenin-7-glucuronide in the AP was conducted by comparing the peak area at 320 nm with a calibration curve developed from the reference standard, as detailed in the Materials and Methods section. The calibration curve demonstrated excellent linearity, with a correlation coefficient (*r*^2^) of 0.9998 (regression equation: *y* = 13.91616x − 8.43512), confirming the method’s reliability. According to this quantification, apigenin-7-glucuronide was identified as a major component of AP, with a content of 9.75 ± 0.02 mg/g (Figure 2).

### Cytotoxicity of AP

Before conducting further experiments, a cytotoxicity assay was performed to determine the appropriate AP concentration range. The viability of A549 cells was evaluated following treatment with AP alone or co-treatment with LPS. AP did not exhibit cytotoxic effects at concentrations up to 100 μg/mL in A549 cells (Supplementary Figure 1). Furthermore, no cytotoxicity was observed when AP was co-treated with LPS at 10 μg/mL. Based on these findings, the maximum concentration of AP used in subsequent *in vitro* assays was set at 100 μg/mL.

### Anti-inflammatory effects of AP

**Figure 3.**
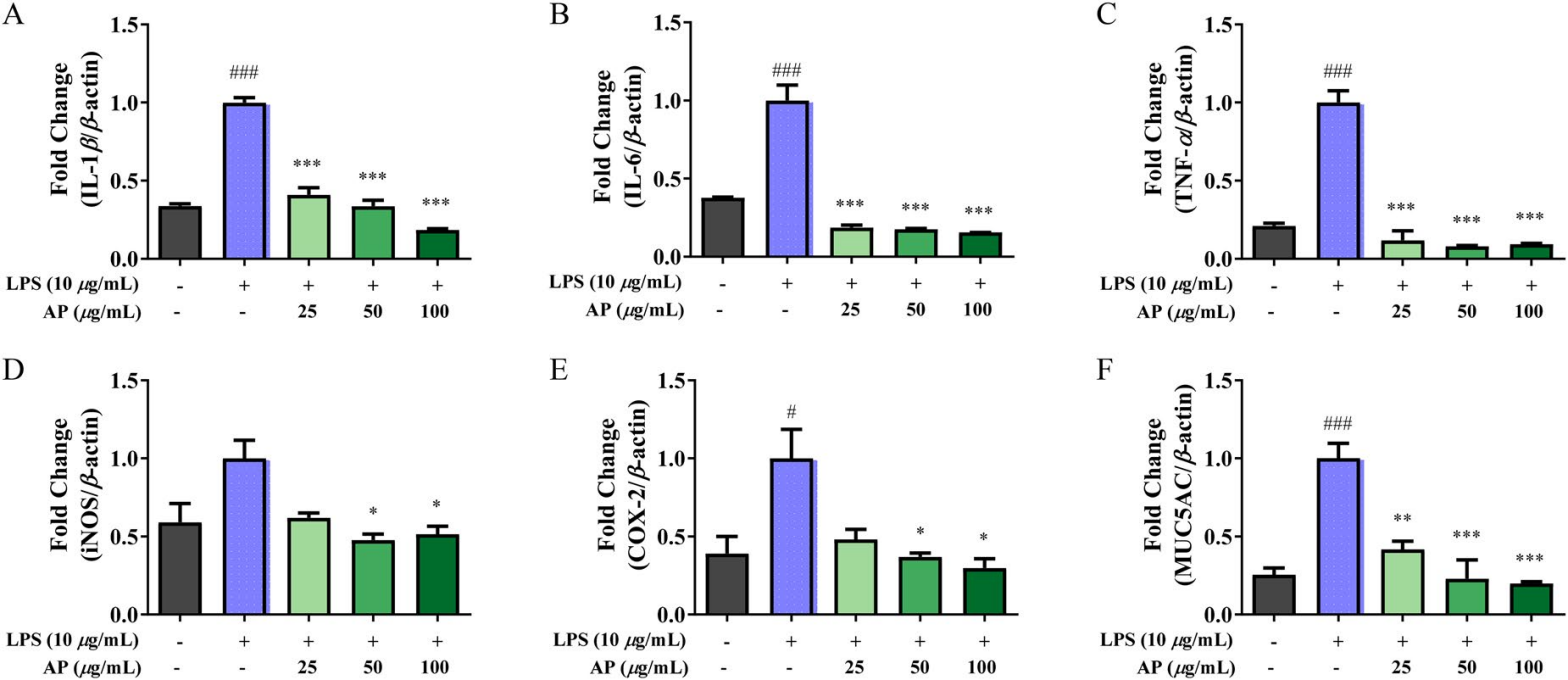
Effects of AP on the mRNA expression of inflammatory cytokines and mediators in LPS-stimulated A549 cells. A549 cells were pretreated with AP for 1 h, followed by LPS stimulation for 24 h. The mRNA expression levels of (A) IL-1β, (B) IL-6, (C) TNF-α, (D) iNOS, (E) COX-2, and (F) MUC5AC were analyzed by qRT-PCR. The data are presented as mean ± SEM (*n* = 3). ^#^*p* < 0.05, ^###^*p* < 0.001 vs. normal control; *p < 0.05, ***p < 0.001 vs. LPS treated control.

As shown in Figure 3, the anti-inflammatory effects of AP were assessed by measuring the mRNA expression levels of pro-inflammatory cytokines (IL-1β, IL-6, TNF-α) and inflammatory mediators (inducible nitric oxide synthase [iNOS] and cyclooxygenase-2 [COX-2]) using qRT-PCR in A549 cells. Treatment with LPS significantly increased expression of all inflammatory markers compared with a control group. In contrast, co-treatment with AP led to a dose-dependent reduction in the expression levels of most inflammatory markers in A549 cells. Notably, in A549 cells, iNOS expression did not show a clear dose-dependent trend; however, significant suppression compared with the LPS control group was observed at 50 and 100 μg/mL. To evaluate the effect of AP on the expression of MUC5AC, a major mucin protein involved in mucus hypersecretion during airway inflammation, LPS stimulation significantly increased expression of MUC5AC mRNA in A549 cells. However, AP treatment dose-dependently suppressed this elevation. Notably, 100 μg/mL of AP showed a comparable level of *MUC5AC* suppression to the DEX-treated positive control. These results demonstrate the anti-inflammatory potential of AP *in vitro.*

### Effects of AP on NF-κB and MAPK signaling pathways in A549 cells

**Figure 4.**
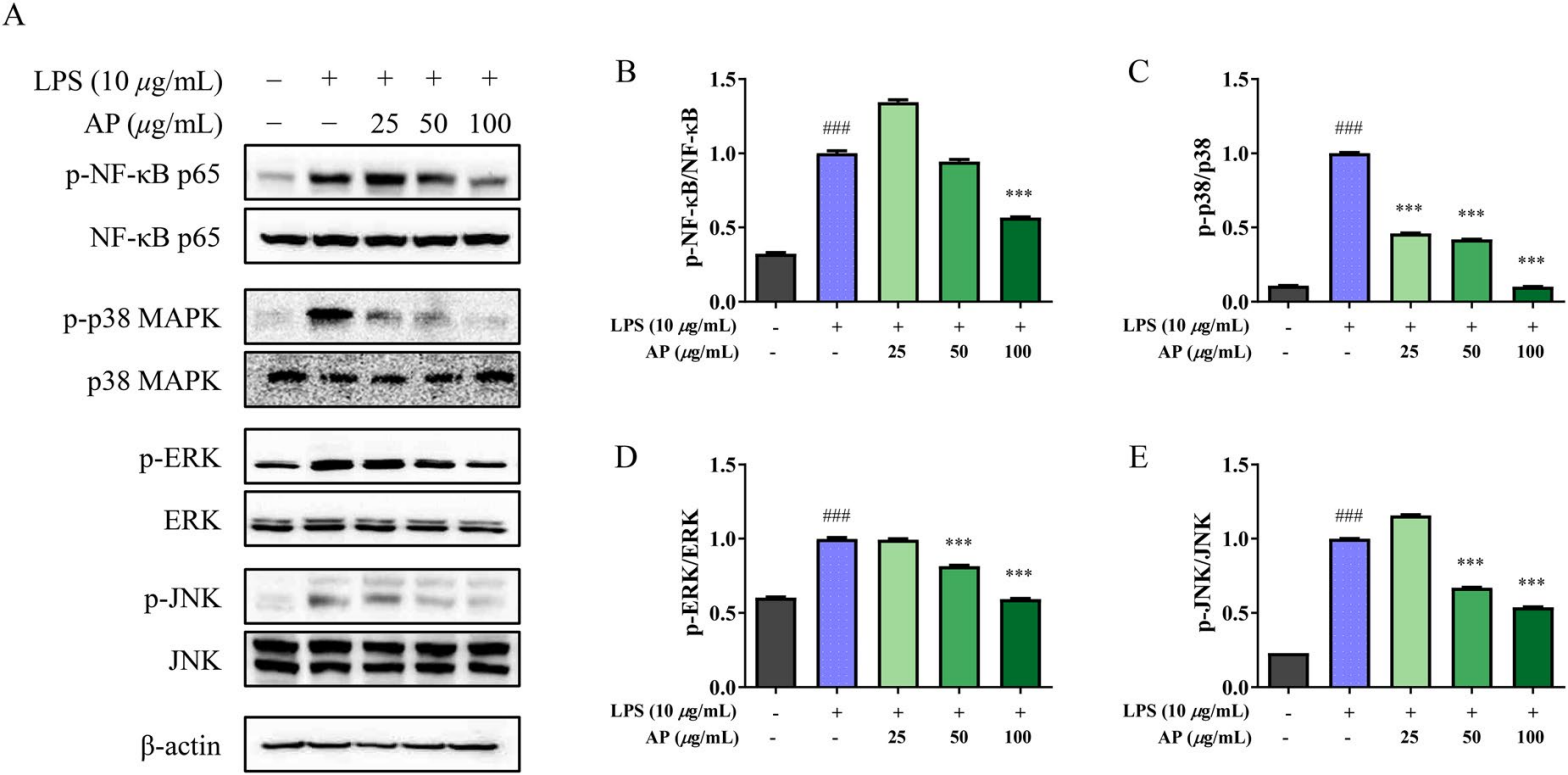
Effects of AP on the NF-κB and MAPK signaling pathway in A549 cells. Cells were pretreated with AP for 1 h, followed by LPS stimulation for 6 h. Protein expression levels were analyzed by Western blots. Phosphorylation levels of NF-κB, p38, ERK, and JNK were evaluated to assess activation of the NF-κB and MAPK signaling pathways. β-actin was used as a loading control. Densitometric analysis of the phosphorylated proteins was normalized to total protein levels and is presented as bar graphs. The data are presented as ^###^*p* < 0.001 vs. normal control; ***p < 0.001 vs. LPS treated control.

After inducing inflammation in A549 cells with LPS, the protein expression levels of phosphorylated and total forms of NF-κB and MAPK (p38, ERK, and JNK) were measured by western blot analysis. As shown in Figure 4, the phosphorylation levels of NF-κB, p38, ERK, and JNK were significantly increased in the LPS-stimulated group, whereas treatment with AP suppressed phosphorylation in a dose-dependent manner.

### Toxicity assessment of AP in the OVA+LPS-induced mouse model

**Figure 5.**
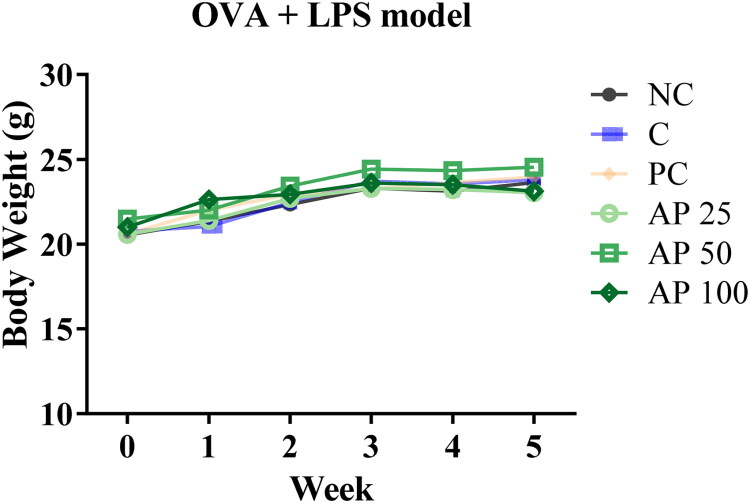
Effect of AP on body weight changes in OVA+LPS-induced mice model. The data are presented as mean ± SEM (*n* = 5). Statistical significance is denoted as ^#^*p* < 0.05 for comparisons with the normal control group, and *p < 0.05 for comparisons with the C (OVA+LPS-induced control group).

As shown in Figure 5, and Table 2, weekly measurements of body weight revealed no significant differences among the experimental groups. Similarly, there were no significant differences in the weights of major organs between groups. Serum biochemical analyses of liver and kidney function indicators demonstrated no evidence of toxicity associated with either the AP or DEX treatment (Table 3). These findings suggest that oral administration of AP is safe and dose not induce systemic toxicity under the conditions of the OVA+LPS-induced airway inflammation model.

**Table 2.** Effects of AP on relative organ weights in an OVA+LPS-induced mouse model.

	Heart	Liver	Lung	Spleen	Kidney	Thymus
NC	0.52 ± 0.05	4.94 ± 0.48	1.05 ± 0.22	0.33 ± 0.03	1.56 ± 0.12	0.15 ± 0.01
C	0.52 ± 0.04	4.61 ± 0.32	1.07 ± 0.20	0.37 ± 0.02	1.46 ± 0.04	0.17 ± 0.33
PC	0.54 ± 0.05	4.41 ± 0.40	1.08 ± 0.23	0.32 ± 0.01	1.56 ± 0.03	0.10 ± 0.04
AP25	0.53 ± 0.02	4.47 ± 0.29	1.19 ± 0.14	0.38 ± 0.04	1.43 ± 0.08	0.15 ± 0.03
AP50	0.52 ± 0.05	4.82 ± 0.17	1.11 ± 0.04	0.37 ± 0.02	1.46 ± 0.07	0.14 ± 0.01
AP100	0.54 ± 0.04	4.21 ± 0.20	1.19 ± 0.07	0.37 ± 0.02	1.46 ± 0.06	0.14 ± 0.06

Values are expressed as mean ± SEM (*n* = 5). ^#^*p* < 0.05 versus NC group, *p < 0.05 versus C (OVA+LPS-induced control) group.

**Table 3.** Effects of AP on serum lipid and liver function indices in an OVA+LPS-induced model.

	NC	C	PC	AP25	AP50	AP100
TG (mg/dL)	191.00 ± 56.14	162.80 ± 27.15	102.80 ± 21.03	146.20 ± 63.02	163.20 ± 8.47	133.40 ± 17.98
Total-C (mg/dL)	188.80 ± 13.85	192.60 ± 17.11	190.00 ± 19.82	191.20 ± 18.14	176.60 ± 8.88	161.60 ± 2.07
HDL-C (mg/dL)	91.00 ± 5.66	87.80 ± 6.22	92.60 ± 7.77	90.60 ± 6.68	83.80 ± 3.96	86.80 ± 2.77
LDL-C (mg/dL)	8.80 ± 0.45	11.20 ± 1.92	10.80 ± 1.79	12.00 ± 1.87	9.60 ± 0.55	10.80 ± 3.03
AST (U/L)	238.75 ± 58.46	356.75 ± 48.13	164.50 ± 39.00	134.75 ± 7.23	142.75 ± 16.35	150.00 ± 9.58
ALT (U/L)	169.75 ± 28.25	331.75 ± 63.897	191.00 ± 26.26	177.00 ± 14.12	198.50 ± 57.11	188.25 ± 57.67
ALP (U/L)	162.40 ± 11.43	128.80 ± 11.82	133.20 ± 12.91	128.8 ± 13.52	132.00 ± 10.37	127.80 ± 12.30
BUN (mg/dL)	32.80 ± 2.95	32.80 ± 7.46	27.00 ± 3.24	27.20 ± 8.93	23.40 ± 0.89	23.60 ± 2.30

ALP, alkaline phosphatase; BUN, blood urea nitrogen; TG, triglyceride; Total-C; Total-cholesterol; HDL-C, high-density lipoprotein cholesterol; LDL-C, low-density lipoprotein cholesterol. Values are expressed as mean ± standard error of the mean (*n* = 5). ^#^*p* < 0.05 versus NC group, *p < 0.05 versus C (OVA+LPS-induced control) group.

### Anti-inflammatory effect of AP in the OVA+LPS-induced mouse model

**Figure 6.**
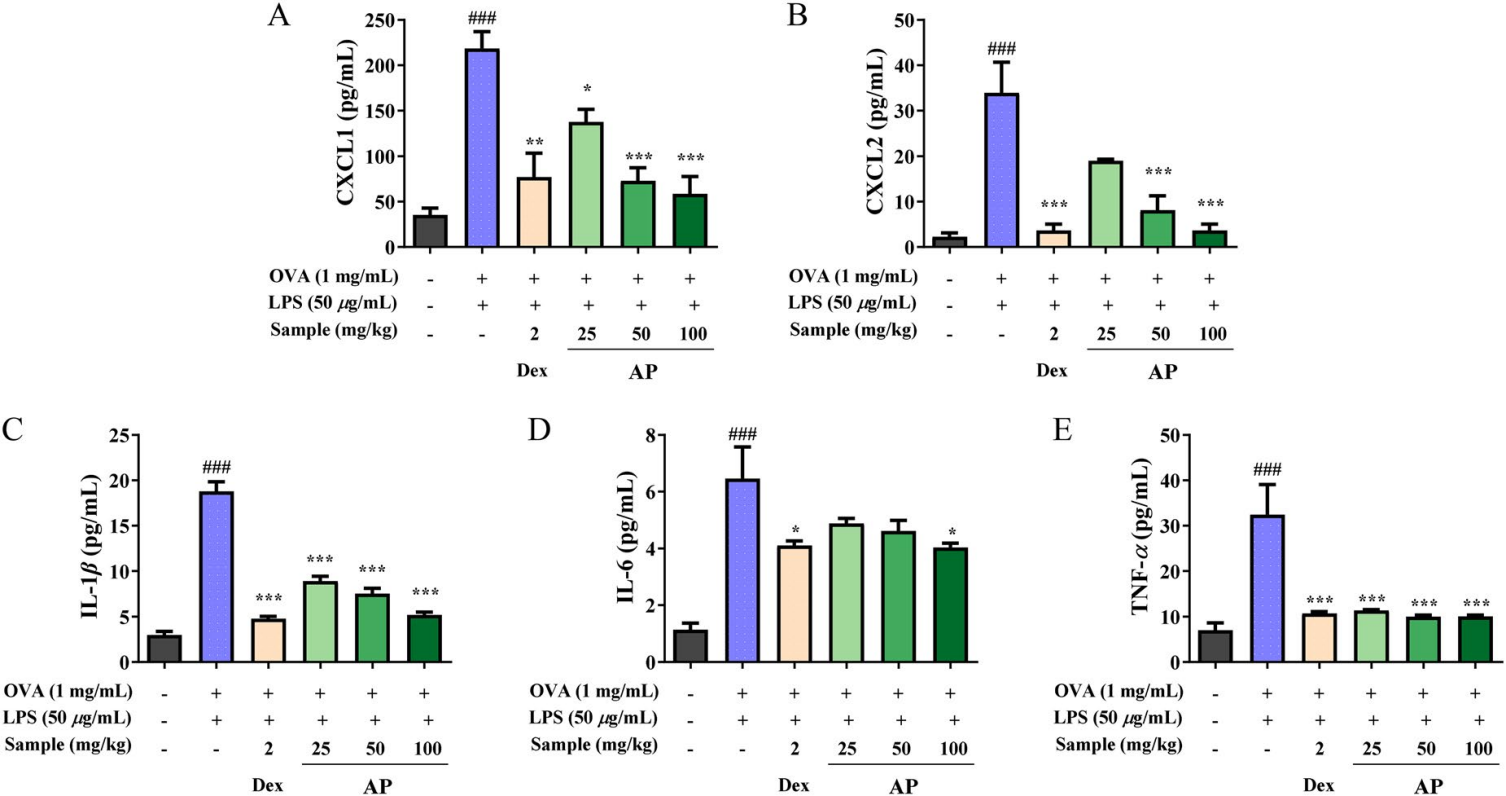
Effect of AP on inflammatory cytokine and chemokine in OVA+LPS-induced mice model. Levels of CXCL1, CXCL2, IL-1β, IL-6, and TNF-α in BALF were analyzed using ELISA. The data are presented as mean ± SEM (*n* = 5). Statistical significance is denoted as ^###^*p* < 0.001 for comparisons with the normal control group, and *p < 0.05, **p < 0.01, ***p < 0.001 for comparisons with the C (OVA+LPS-induced control group).

To evaluate the anti-inflammatory effects of AP *in vivo*, the levels of CXCL-1, CXCL-2, IL-1β, IL-6, and TNF-α were measured in bronchoalveolar lavage fluid (BALF). As demonstrated in Figure 6, AP significantly reduced the levels of CXCL-1 and CXCL-2, with statistically significant reductions observed in the 50 and 100 mg/kg groups compared to the OVA+LPS-induced control group. Notably, CXCL-1 and CXCL-2 decreased to approximately 73.2% and 89.2% of the levels in the OVA+LPS group at a dosage of 100 mg/kg (Figure 6(A,B)). The levels of IL-1β were also diminished, reaching statistical significance at 100 mg/kg, with a suppressive effect comparable to that of the positive control. Specifically, IL-1β was reduced by approximately 72.5% relative to the OVA+LPS group at 100 mg/kg (Figure 6(C)). Regarding IL-6, a declining trend was observed as the dose of AP increased; however, statistical significance was only achieved at 100 mg/kg. Consistently, IL-6 exhibited an approximate reduction of 37.4% at 100 mg/kg (Figure 6(D)). Importantly, TNF-α levels were markedly suppressed across all AP-treated groups (25–100 mg/kg), with effects approaching those observed in the dexamethasone (DEX)-treated positive control group. Specifically, TNF-α levels were decreased by approximately 65.0% to 69.2% across the varying AP doses compared to the OVA+LPS group (Figure 6(E)). In summary, these findings demonstrate that AP exerts potent anti-inflammatory effects within the OVA+LPS-induced airway inflammation model by downregulating key pro-inflammatory cytokines and chemokines present in BALF.

### Lung-protective effect of AP in the OVA+LPS-induced mouse model

**Figure 7.**
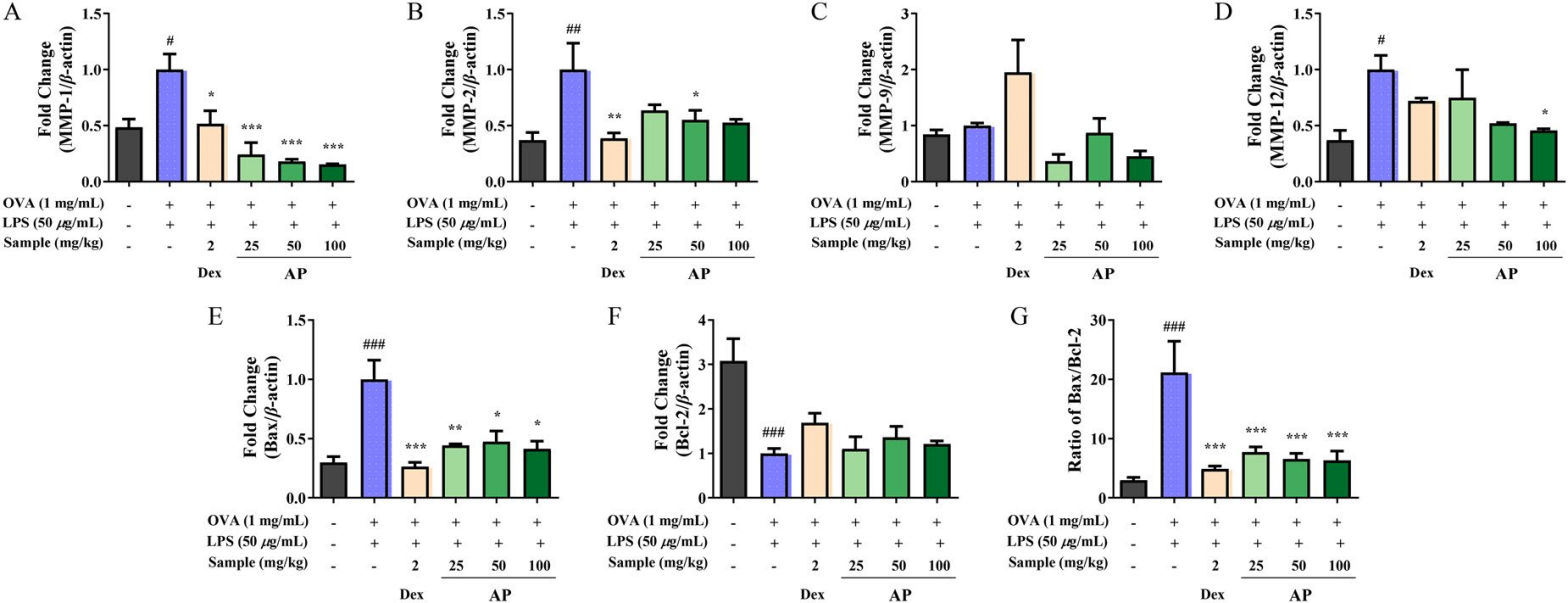
Effect of AP on mRNA level in OVA+LPS-induced mice model. (A–F) Gene expression of MMP-1, MMP-2, MMP-9, MMP-12, Bax, and Bcl-2 was measured in OVA+LPS-induced mice lung tissue using qRT-PCR analysis. (G) Changes in the Bax/Bcl-2 ratio in OVA+LPS-induced mice lung tissue. The data are presented as mean ± SEM (*n* = 5). Statistical significance is denoted as ^##^*p* < 0.01 and ^###^*p* < 0.001 for comparisons with the normal control group, and *p < 0.05, **p < 0.01, and ***p < 0.001 for comparisons with the C (OVA+LPS-induced control group).

To investigate the protective effects of AP on lung tissue, we assessed the mRNA activity levels of MMP genes, including those that express MMP-1, −2, −9, and −12 in lung tissue from the OVA+LPS-induced mouse model. As shown in Figure 7, expression of MMP-1 was reduced in a dose-dependent manner following AP treatment, with significant suppression observed at a high dose, which was even greater than that of the positive control. Notably, MMP-1 expression at 100 mg/kg was reduced to approximately 84.7% of that in the OVA+LPS group (Figure 7(A)). Similarly, AP treatment led to significant dose-dependent reductions in the expression of MMP-9 and MMP-12. At 100 mg/kg, MMP-9 and MMP-12 were reduced by approximately 54.9% and 54.5%, respectively, compared with the OVA+LPS group (Figure 7(C,D)). Notably, MMP-12 expression was significantly suppressed even at the lowest AP dose, showing greater efficacy than the positive control group. In contrast, MMP-2 expression decreased with AP treatment; however, the changes were not statistically significant, and no significant difference was observed between the OVA+LPS control and normal control groups (Figure 7(B)). In addition, AP treatment resulted in a dose-dependent reduction of the Bax/Bcl-2 ratio in lung tissue, as shown in Figure 7(G). Starting from the mid-dose group, Bcl-2 expression was significantly upregulated, and Bax expression was downregulated, indicating an anti-apoptotic effect that surpassed that of the positive control. Consistent with these findings, the Bax/Bcl-2 ratio decreased to approximately 70.0% of the OVA+LPS group at 100 mg/kg (Figure 7(G)).

**Figure 8.**
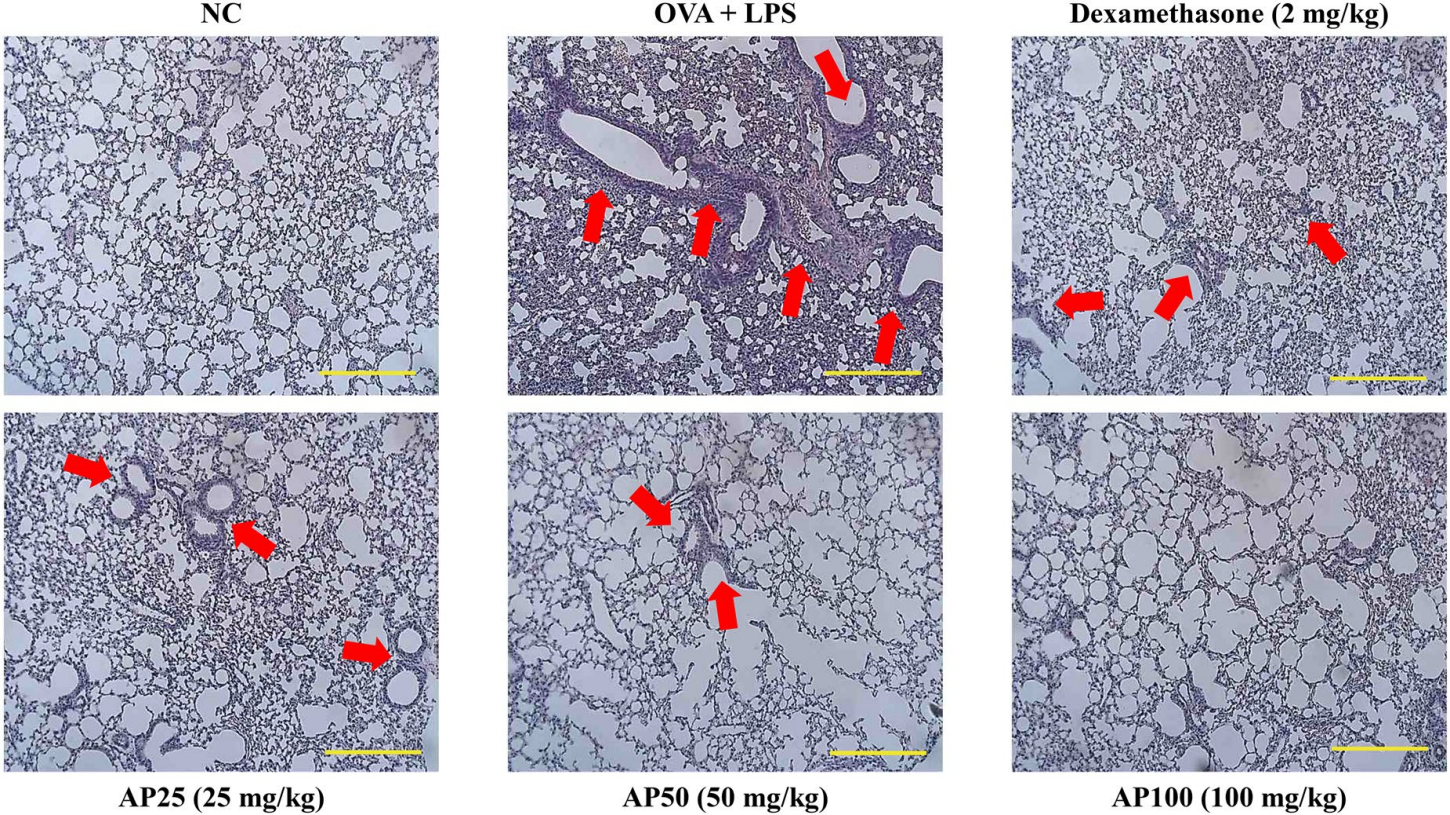
Effect of AP on histopathological changes in OVA+LPS-induced mice lung tissue. Histopathological changes in the lung tissues of inflammation-induced mice by OVA+LPS with administration of dexamethasone and AP. Magnification, ×100; scale bar, 100 μm.

Histopathological evaluation of lung tissues is shown in Figure 8. OVA+LPS stimulation induced prominent thickening of the bronchial wall and marked inflammatory-cell infiltration, indicating more severe allergic and inflammatory responses compared with those observed in the OVA-only control group. In contrast, treatment with either AP or the positive control resulted in a notable reduction in bronchial wall thickness and inflammatory-cell infiltration, with lung histologies comparable to those of the normal control group.

## Discussion

Respiratory diseases such as asthma and COPD are primarily associated with inflammation in the bronchi and lung parenchyma (Tian et al. 2019). Oxidative stress-induced inflammation activates key signaling pathways, including NF-κB and MAPK, leading to the production of pro-inflammatory cytokines (Mishra et al. 2018; Abohassan et al. 2022). Alveolar macrophages secrete mediators such as TNF-α, interleukins, and CXCL chemokines, which recruit neutrophils and sustain chronic inflammation (Fiorentino et al. 1991). Additionally, activation of alveolar macrophages and neutrophils stimulates the expression of MMPs, increasing proteolytic activity and contributing to the degradation of lung tissue (Corbel et al. 2000). Apoptosis of lung cells further exacerbates tissue damage. As inflammation and cell death progress, patients experience symptoms such as sputum production, dyspnea, and chest tightness.

While corticosteroids are the most effective anti-inflammatory agents for these conditions, their long-term use produces significant systemic side effects. (Abdelaziz et al. 2018; Moon et al. 2021). Consequently, there is growing interest in natural compounds with both potent bioactivity and low toxicity as alternative therapeutic options.

A traditional medicinal herb, AP causes anti-inflammatory effects by downregulating cytokine expression in LPS-stimulated RAW 264.7 macrophages, as well as mucosal protective effects in animal models of gastritis (Jin et al. 2016). However, its efficacy in respiratory inflammation had not yet been investigated. This study was designed to evaluate the anti-inflammatory and protective effects of AP in a mouse model of airway inflammation induced by OVA+LPS, while also exploring its potential as a plant-based candidate for respiratory inflammation.

An MTT assay was performed using A549 human alveolar epithelial cells to assess the non-cytotoxic concentrations of AP. The results showed no cytotoxicity at concentrations of 25, 50, and 100 μg/mL (Figure 2). A549 cells are derived from human lung adenocarcinoma and may not fully recapitulate the differentiation status, barrier properties, and inflammatory responses of normal human airway epithelium (Stewart et al. 2012; Wiese-Rischke et al. 2021). In contrast, primary airway epithelial cells are limited in availability, have a finite lifespan in culture, and show donor-to-donor variability (Martinovich et al. 2017). Therefore, we chose A549 cells as a practical, standardized epithelial model for the mechanistic screening of anti-inflammatory effects, with high reproducibility in this study.

IL-1β, IL-6, and TNF-α are pro-inflammatory cytokines, while COX-2 and iNOS are inflammation-related enzymes associated with the production of nitric oxide (Peters et al. 2016; Quoc et al. 2021). To evaluate the anti-inflammatory effect of AP, A549 cells were treated with AP (25, 50, and 100 μg/mL) and LPS (10 μg/mL), followed by qRT-PCR analysis. AP treatment significantly suppressed the mRNA expression of IL-1β, IL-6, TNF-α, COX-2, and iNOS in A549 cells (Figure 3). Several studies have demonstrated that LPS is a potent inducer of MUC5AC expression, and it is therefore used widely (Wang et al. 2009; Hardaker et al. 2010; Di Lorenzo et al. 2015; Maldonado et al. 2016). In this study, we also investigated the effect of AP on MUC5AC, a major mucin gene involved in mucus hypersecretion during inflammation of the airway. LPS stimulation significantly increased mRNA expression of MUC5AC in A549 cells, whereas AP treatment dose-dependently suppressed this elevation. Notably, the 100 μg/mL concentration of AP produced a level of MUC5AC suppression comparable to that of the DEX-treated positive control. These results demonstrate the anti-inflammatory potential of AP *in vitro*.

NF-κB and MAPK pathways play important roles in inflammation and cell regulation (Duan et al. 2021). Western blot analysis was performed to investigate the anti-inflammatory mechanism of AP. A549 cells were treated with AP (25, 50, and 100 μg/mL) and LPS (10 μg/mL), and the expression levels of NF-κB p65 and p38 ERK, and JNK MAPKs were assessed. AP treatment significantly reduced the phosphorylation levels of these signaling proteins (Figure 4). Although these results imply the involvement of NF-κB and MAPK pathways, the study does not establish a direct causal relationship. Further investigation using pathway-specific inhibitors and genetic techniques is required to determine whether inhibition of NF-κB/MAPK is the primary mechanism or a secondary effect of earlier suppression of inflammatory mediators.

For *in vivo* evaluation, male BALB/c mice were randomly classified in the OVA+LPS-induced asthma model according to guidelines set by the Korean Ministry of Food and Drug Safety. Body weight was monitored weekly to assess the effects of the treatments on systemic toxicity. No significant differences in body weight were observed between the groups in all models (Figure 5). Similarly, the organ-to-body-weight ratios showed no significant differences among the groups (Table 2).

Serum levels of cholesterol (CHOL), triglyceride (TG), high-density lipoprotein (HDL), and low-density lipoprotein (LDL) are common indicators of lipid metabolism and liver tissue damage. Alkaline phosphatase (ALP) reflects liver and gallbladder function, while blood urea nitrogen (BUN), a final product of protein metabolism, is an indicator of liver function (Lee et al. 2013). To evaluate the effect of the treatment on liver function, serum levels of CHOL, TG, HDL, LDL, ALP, and BUN were measured. No significant differences were observed among the groups in the OVA+LPS-induced mouse models (Table 3).

BALF, collected from the bronchial tubes using ice-cold PBS, contains various immune cells and inflammatory mediators. To evaluate airway inflammation, levels of IL-1β, IL-6, CXCL-1, CXCL-2, and TNF-α in BALF were measured using ELISA kits (Su et al. 2011). In the OVA+LPS model, OVA and LPS significantly increased the levels of IL-1β, CXCL-1, CXCL-2, and TNF-α, which were dose-dependently reduced by AP treatment (Figure 6).

MMPs are proteolytic enzymes that contribute to lung tissue damage (Wang et al. 2009). To assess the inhibitory effect of AP on MMP expression, qRT-PCR amplification was performed using cDNA synthesized from RNA extracted from lung tissue. AP administration significantly reduced the mRNA expression of MMP-9 and MMP-12 (Figure 7).

Bax regulates the release of cytochrome c from mitochondria and plays a key role in the apoptotic pathway. The Bax/Bcl-2 ratio is positively correlated with the degree of apoptosis (Tian et al. 2019). To evaluate the effect of AP on apoptosis, qRT-PCR amplification was performed using cDNA synthesized from lung tissue RNA. AP treatment significantly reduced the Bax/Bcl-2 mRNA ratio in the OVA+LPS-induced mice (Figure 7).

H&E staining was used to assess the histopathological changes in lung tissues. Inflammation induced by OVA+LPS resulted in cellular swelling, tissue lesions, and excessive infiltration of inflammatory cells (Figure 8). These pathological changes were alleviated by treatment with AP or DEX.

Several limitations of this study should be noted. Although the short-term OVA+LPS protocol provided initial insights into its anti-inflammatory and protective effects *in vivo*, the small sample size (*n* = 5 per group) warrants validation in larger cohorts to enhance reproducibility and applicability. Additionally, since the pharmacokinetic and bioavailability profiles of oral AP were not evaluated, future research should clarify exposure-response relationships and optimal dosing strategies. Furthermore, as the study lacks human data and relies on primary airway epithelial models, these results need to be confirmed in human-relevant systems and ultimately in clinical trials to ensure their translational significance.

## Conclusions

In conclusion, a water extract of AP exhibited anti-inflammatory effects by inhibiting the mRNA expression of IL-1β, IL-6, TNF-α, iNOS, COX-2, and MUC5AC in A549 cells. These effects were associated with the suppression of NF-κB and MAPK signaling pathways. *In vivo*, AP reduced the expression of MMP-9 and MMP-12, as well as the Bax/Bcl-2 ratio, indicating anti-proteolytic and anti-apoptotic activity, without inducing toxicity. It also significantly reduced levels of pro-inflammatory cytokines, such as CXCL-1, CXCL-2, IL-1β, IL-6, and TNF-α, in BALF.

These findings suggest that AP, through alleviating airway inflammation and protecting lung tissue from damage, is a promising plant-derived candidate for further investigation in inflammatory airway diseases. Future research should verify these results using longer-term models of chronic airway inflammation and, ultimately, through clinical trials to establish its translational and clinical potential.

## Supplementary Material

Supplementary_data_Pharmaceutical_Biology_260109.docx

## Data Availability

The data that support the findings of this study are available from the corresponding author upon reasonable request.
