# Supplementary material for: Mechanistic insight into the anti-inflammatory and lung-protective effects of Agrimonia pilosa extract via NF-κB/MAPK inhibition in ovalbumin- and lipopolysaccharide-induced respiratory inflammation models
Source: Pharm Biol. 2026 Mar 1;64(1):363–78. doi: 10.1080/13880209.2026.2627661 (PMC12954814; doi:10.1080/13880209.2026.2627661)
Supplement: Supplementary_data_Pharmaceutical_Biology_260109.docx [file IPHB_A_2627661_SM4099.docx]

**SUPPLEMENTARY DATA**

**Mechanistic insight into the anti-inflammatory and lung-protective effects of *Agrimonia pilosa* extract via NF-κB/MAPK inhibition in ovalbumin- and lipopolysaccharide-induced respiratory inflammation models**

Yeong-Geun Lee^a,b^, Jeong Eun Kwon^a,b^, Dae Won Park^a,b^, Hae Rim Lee^a^, Jinhyuk Lee^c^, Yong-Min Choi^c^, Eun-Ji Cho^c^, Se Chan Kang^a,b,d,*^

^a^Department of Oriental Medicine Biotechnology and Graduate School of Biotechnology, Kyung Hee University, Yongin 17104, Gyeonggi, Republic of Korea

^b^BioMedical Research Institute, Kyung Hee University, Yongin 17104, Republic of Korea

^c^Daesang Wellife, Seoul 03130, Republic of Korea

^d^Research Institute, Mbiometherapeutics Co., Ltd., Yongin 17104, Gyeonggi, Republic of Korea

*Corresponding author:

Se Chan Kang

Department of Oriental Medicine Biotechnology and Graduate School of Biotechnology, Kyung Hee University, 1732 Deogyeong-daero, Giheung-gu, Yongin-si, Gyeonggi-do 17104, Republic of Korea

Phone: +82 31 201 2687

Fax: +82 31 204 8116

E-mail: [sckang@khu.ac.kr](mailto:sckang@khu.ac.kr)

**Supplementary figure legends**

**Supplementary Figure 1**. **Effects of AP on cell viability in A549 cells**. (A) A549 cells were incubated with various concentrations of AP (0–100 μg/mL) for 24 h, and cell viability was measured by MTT assays. (B) A549 cells were treated with different concentration of LPS for 24 h, and cell viability was assessed to determine the optimal LPS concentration for subsequent experiments. (C) A549 cells were pretreated with AP for 1 h, followed by LPS stimulation for 24 h, and cell viability was measured by MTT assays. The data are presented as mean ± SEM (n = 3). ^###^*p* < 0.001 vs. normal control.

**
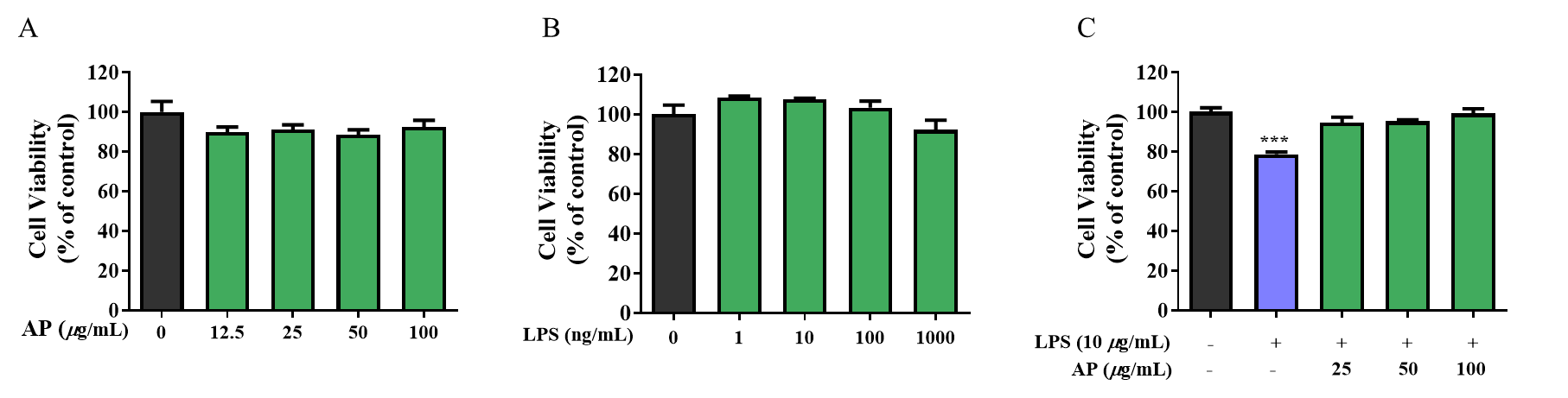
**

**Supplementary Figure 1**.
